# Effects of Hand-Rearing on Reproductive Success in Captive Large Cats *Panthera tigris altaica*, *Uncia uncia*, *Acinonyx jubatus* and *Neofelis nebulosa*

**DOI:** 10.1371/journal.pone.0155992

**Published:** 2016-05-23

**Authors:** Maja Coulthard Hampson, Christoph Schwitzer

**Affiliations:** 1 Bristol Zoological Society, Bristol, United Kingdom; 2 School of Veterinary Sciences, University of Bristol, Bristol, United Kingdom; Federal University of Parana (UFPR)) – Campus Palotina, BRAZIL

## Abstract

Species Survival Plans and European Endangered Species Programmes have been developed for several species of endangered felids in order to build up captive reserve populations and support their conservation in the wild. The Siberian tiger (*Panthera tigris altaica*), snow leopard (*Uncia uncia*), cheetah (*Acinonyx jubatus*) and clouded leopard (*Neofelis nebulosa*) are managed in such *ex situ* conservation programmes. Many zoological institutions hand-rear offspring if rearing by the mother fails. Hand-rearing can cause behavioural problems, resulting in decreased copulation and lower breeding success in some species. In this study, studbook data subsets were examined: from 1901 to 2011; and 2000 to 2011. We analysed records from 4273 Siberian tigers, 2045 snow leopards, 3435 cheetahs, and 804 clouded leopards. We assessed the number of offspring produced, litter size, age at first reproduction, longevity, infant mortality and generational rearing of hand-reared versus parent-reared individuals. Hand-reared Siberian tigers (p<0.01; p = 0.0113), snow leopards (p<0.01), male cheetahs (p<0.01) and female clouded leopards (p<0.01) produced fewer offspring than parent-reared individuals. Hand-reared snow leopard breeding pairs had larger litters than parent-reared pairs (p = 0.0404). Hand-reared snow leopard females reproduced later in life (p<0.01). Hand-reared female Siberian tigers lived shorter lives, while hand-reared cheetahs lived longer (p<0.01; p = 0.0107). Infant mortality was higher in hand-reared snow leopards (p<0.01) and male cheetahs (p = 0.0395) in the 1901–2011 dataset and lower in hand-reared female Siberian tiger and male snow leopard cubs (p = 0.0404; p = 0.0349) in the 2000–2011 dataset. The rearing of the mother and subsequent rearing of offspring showed a significant relationship for all species (p<0.01 for Siberian tiger and snow leopard cubs; p<0.001 for cheetah and snow leopard cubs). Taking into account the limited carrying capacity of zoos, the results of this study highlight that careful consideration should be taken when deciding whether or not to hand-rear individuals that are part of Species Survival Plans and European Endangered Species Programmes.

## Introduction

The survival of several large felid species with very small and highly threatened remnant populations in the wild may ultimately depend on managed captive reserve populations such as those coordinated in Species Survival Plans (SSPs) and European Endangered Species Programmes (EEPs). The Siberian tiger (*Panthera tigris altaica*), snow leopard (*Uncia uncia*), cheetah (*Acinonyx jubatus*) and clouded leopard (*Neofelis nebulosa*) are primarily solitary cats that are threatened in the wild and have captive populations managed as SSPs and EEPs in North America and Europe, respectively [1]. The goal of SSPs and EEPs is to contribute to the conservation of species through cooperatively managed *ex situ* breeding programmes. Additionally, these programmes aim to form healthy, self-sustaining, genetically diverse populations of species in human care [1]. This includes research to inform management decisions and determine best breeding practices for a particular species. However, in such programmes, many participating institutions hand-rear individuals if parent-rearing fails or is not an option, which can lead to stereotypic or abnormal behaviours, failure to establish adequate sexual posturing for copulation, or lack of social-sexual behaviour entirely [2, 3]. Hand-rearing can hence potentially compromise the demographic and genetic management tactics utilized by these conservation plans to establish and maintain self-sustaining populations [4].

Siberian tigers, snow leopards, cheetahs and clouded leopards are primarily solitary cats. Siberian tigers occupy territories in the Russian Far East. Snow leopards inhabit the mountains of Central and South Asia. Cheetahs are African cats, well known as the world's fastest land animal. Females are solitary but males often live together in coalitions. Little is known about the biology of clouded leopards due to their secretive nature. They reside in the Himalayan foothills through mainland Southeast Asia and into China. Cubs of these species rely on their mother's milk to the ages of three to six months [5, 6, 7, 8]. Once the cubs are weaned they stay with their mothers for protection, learning hunting and social skills until adulthood. All of these species are threatened by illegal hunting, habitat loss, loss of prey, and persecution [1].

Proper socialization is essential for large felids to develop the correct behavioural repertoire for mating and copulation. For this reason, hand-rearing carnivores can present challenges with regards to the development of normal behaviour [9]. Proper socialization can take up to three years in carnivores [10, 11]. Failure to socialize during development can result in fear or aggression around conspecifics, lack of conspecific play, difficulty reproducing and a fixation on humans [12]. These psychological disorders can lead to incompatibility with conspecifics, lack of breeding, and inappropriate care of infants [13, 14, 15, 16]. Parental rearing can have nutritional, developmental and behavioural benefits for the offspring [9, 17]. However, situations can arise in captivity where staff feel that it is necessary to hand-rear a cub in order to ensure its survival. Hand-rearing can result, for example, from high levels of intervention during the first weeks post-partum, abandonment or an undetected pregnancy which may cause the mother to give birth in an unsuitable location [5, 10, 11].

The majority of studies on the effects of hand-rearing in relation to reproductive success have focused on non-human primates. For example, hand-reared western lowland gorillas (*Gorilla gorilla gorilla*) had lower breeding success than mother-reared gorillas and were less likely to breed with other hand-reared individuals [4, 18, 19, 20, 21]. Some studies on carnivores have begun to show that hand-rearing affects their behaviour and reproductive success as well. Green et al. [22] found that hand-reared coyotes (*Canis latrans*) kept in captivity produced fewer offspring than would be expected from wild mother-reared individuals. Both female and male domestic cats (*Felis catus*) raised by humans alone without conspecific contact copulated significantly less than those raised by their mothers alongside conspecifics [23, 24]. Additionally, female cats raised by humans displayed extreme aggression toward both male conspecifics and their human caretakers.

Research on non-human primates has suggested that it is essential to integrate hand-reared infants into a diverse group of conspecifics as early as possible [25, 26]. As parental rearing positively impacts future reproductive success of gorilla offspring, the gorilla SSP is placing a strong emphasis on the management protocols that encourage maternal competence before and after the actual birth of an infant [4]. There is a reproductive skew present in gorillas due to rearing type [4, 18, 19, 20, 21] and evidence of a similar trend in domestic cats as well [23]. Research has shown that hand-reared gorillas have fewer opportunities to reproduce. Likewise, hand-reared domestic cats copulate significantly less than parent-reared individuals. For gorillas, it is unclear whether the lack of reproductive opportunity is due to an individual's behaviour within a group of conspecifics or artificial captive breeding management; however, Mellen's study [23] illustrates that hand-reared domestic cats copulate less due to behaviour towards conspecifics and physical positioning and posture during copulation.

Over the past few decades, zoos have become major players in global species conservation. SSPs and EEPs aim to help conserve endangered species through cooperatively managed breeding programs within zoological institutions, thus establishing captive reserve populations. Without comprehensive research on the outcomes of hand-rearing large carnivores, zoos may be ill-equipped to implement adequate infant rearing practices for the benefit of SSPs and EEPs. Therefore, the aim of this study was to investigate the reproductive success of hand-reared felids compared to that of parent-reared individuals by comparing the number of offspring produced, litter size, age at first reproduction, longevity, infant mortality and next generation rearing patterns between groups.

## Materials and Methods

Data organization was carried out in June and July of 2014. All data were derived from the 2011 international studbooks SPARKS dBase files. In order to account for potential improvements in captive husbandry practices over the period of analysis, the data were analysed in two subsets: the totality of the data ranging from 1901 to 2011, and a subset ranging from 2000–2011. In total, we analysed records from 4273 Siberian tigers, 2045 snow leopards, 3435 cheetahs, and 804 clouded leopards. ELT-tools Database Browser version 5.1.0.7 was used to organize the data (S1 Table. SQL query for number of offspring, age at first reproduction, longevity, infant mortality, and generational rearing; S2 Table. SQL query for litter size). The number of offspring, litter size, age at first reproduction, age at death, occurrence of infant mortality and dam rearing were isolated using Structured Query Language (SQL)–a programming language for expressing complex consolidation and collation of data. Individuals that were not assigned a standard gender (0 = female; 1 = male) or rearing value (P = parent-reared; H = hand-reared) were excluded from the data set. Between all four species of large felid, 21.5–49.5% of the total data set were excluded due to these parameters. Ultimately, 1173 Siberian tigers, 823 snow leopards, 3499 cheetahs, and 543 clouded leopards were not included in this study. Separate comparisons were made between hand-reared and parent-reared females and males. Ethical approval was obtained from the Ethical Review Group at Bristol University. All data were tested for normalcy using a Kolmogorov-Smirnov Statistical tests were conducted using JMP 11.

### Number of offspring

The number of offspring was determined by counts of the number of individuals a particular male had sired or female had birthed during their studbook lifetime. For the 1901–2011 data analysis, 1948 females and 1857 males were included out of 4273 Siberian tigers. Of 2045 snow leopards, 938 females and 900 males were included. Of 3435 cheetahs, 1358 females and 1473 males were included. Of 804 clouded leopards, 253 females and 275 males were included in the analysis. For the 2000–2011 data analysis, all 418 Siberian tigers were included, 230 females and 188 males. Of 480 snow leopards, 261 females and 219 males were included. Of 905 cheetahs, 430 females and 475 males were included. Of 149 clouded leopards, 81 females and 68 males were included in the analysis. Individuals that did not produce any offspring were excluded from this analysis. Data for all species were normally distributed and a t-test was used to determine statistical significance.

### Litter Size

The litter size was determined by the number of individuals born to each dam and each sire during a single reproductive event. Individuals that did not produce any offspring were excluded from this analysis. The data were sorted by female-male reproductive pairs as follows: hand-reared female and hand-reared male (H-H); hand-reared female and parent-reared male (H-P); parent-reared female and hand-reared male (P-H); and parent-reared female and parent-reared male (P-P). For the 1901–2011 dataset, 2045 Siberian tiger, 1074 snow leopard, 1102 cheetah, and 440 clouded leopard pairs were analysed. For the 2000–2011 dataset, 418 Siberian tiger, 326 cheetah, 283 snow leopard, and 83 clouded leopard pairs were analysed. Litters were treated as individual reproductive events and not grouped based on how many litters a single individual produced. Data for all species were normally distributed. A one-way ANOVA was used to determine differences between pairing groups, followed by a Tukey's HSD post-hoc test to determine which groups differed.

### Age at first reproduction

Age at first reproduction was determined by subtracting an individual’s date of birth from the date of birth of their first offspring. Individuals that gave birth to stillborn young or to offspring that died shortly after birth were included. Individuals that did not produce any offspring were excluded from this analysis. Different tests were required for the analysis of female and male age at first reproduction because the data for females were normally distributed while the data for males were not. For the 1901–2011 dataset, 466 female and 397 male Siberian tigers, 324 female and 284 male snow leopards, 260 female and 226 male cheetahs, and 50 female and 45 male clouded leopards were included in this analysis. For the dataset spanning 2000–2011, 36 female Siberian tigers, 26 female and 47 male snow leopards, 47 female and 36 male cheetahs and 11 female and 10 male clouded leopards were included. Analysis for male Siberian tigers could not be carried out as no hand-reared males produced any offspring. The data for Siberian tigers, snow leopard females, cheetahs and clouded leopards were normally distributed and we used a t-test to determine statistical differences. A Kolmogorov-Smirnov test showed that the data for 1901–2011 snow leopard males were not normally distributed, therefore a Mann-Whitney U test was used.

### Longevity

The date of death minus the date of birth was used to determine longevity. Individuals that were “lost to follow up” did not have a recorded date of death and were excluded from the analysis. Individuals that died within the infant mortality age range for their species were also excluded from the analysis. Infant mortality is defined in the *infant mortality* section below. For the 1901–2011 analysis, 731 female and 632 male Siberian tigers, 450 female and 433 male snow leopards, 729 female and 769 male cheetahs, and 112 female and 120 male clouded leopards were included. For the 2000–2011 analysis, 22 female and 17 male Siberian tigers, 18 female and 16 male snow leopards, 64 female and 70 male cheetahs, and 4 female and 5 male clouded leopards were included. Data for most species were normally distributed and a t-test was used to determine statistical significance. A Kolmogorov-Smirnov test showed that data for 2000–2011 cheetahs were not normally distributed, therefore a Mann-Whitney U test was used.

### Infant mortality

Infant mortality was defined as death before weaning age. Siberian tiger cubs are dependent solely upon their mothers for nourishment for up to four months [27]. Snow leopards and cheetahs are weaned at three months [6, 7]. Clouded leopard cubs are weaned at 100 days [8]. For the 1901–2011 dataset, 1234 female and 1154 male Siberian tigers, 690 female and 678 male snow leopards, 994 female and 1060 male cheetahs, and 192 female and 205 male clouded leopards were included in the analysis. For the 2000–2011 dataset, 36 female and 46 male Siberian tigers, 84 female and 67 male snow leopards, and 74 female and 74 male clouded leopards were included in the analysis. The data for Siberian tigers, cheetahs and clouded leopards were normally distributed and we used a t-test to determine statistical differences. A Kolmogorov-Smirnov test showed that data for 1901–2011 snow leopards were not normally distributed, therefore a Mann-Whitney U test was used.

### Generational rearing

Since Siberian tigers, snow leopards, cheetahs and clouded leopards have maternal care only [8, 28, 29, 30], data on dam rearing type were paired with the rearing type of their offspring. For the 1901–2011 dataset, we analysed 4273 Siberian tigers, 2045 snow leopards, 3435 cheetahs, and 804 clouded leopards. For 2000–2011, we analysed 418 Siberian tigers, 480 snow leopards, 905 cheetahs, and 149 clouded leopards. A Fisher's exact test was used to determine statistical significance between rearing types of dams and the subsequent rearing of their offspring.

## Results

### Siberian tiger

#### 1901–2011

**Fig 1:**
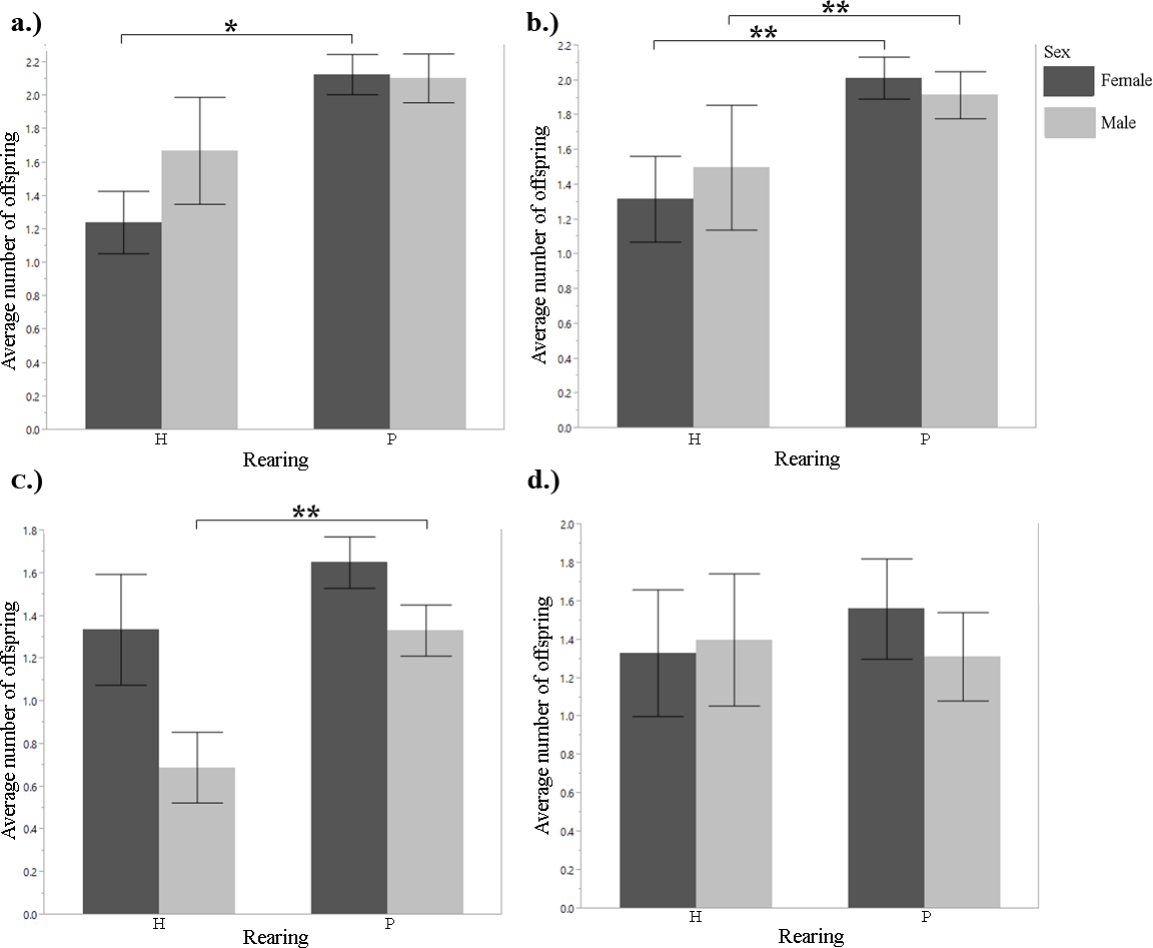
Mean number of offspring a female has birthed or a male has sired during their studbook lifetime, by rearing type (1901–2011). A.) Siberian tiger B.) snow leopard C.) cheetah D.) clouded leopard. t-test: *p<0.05; **p<0.01.

**Fig 2:**
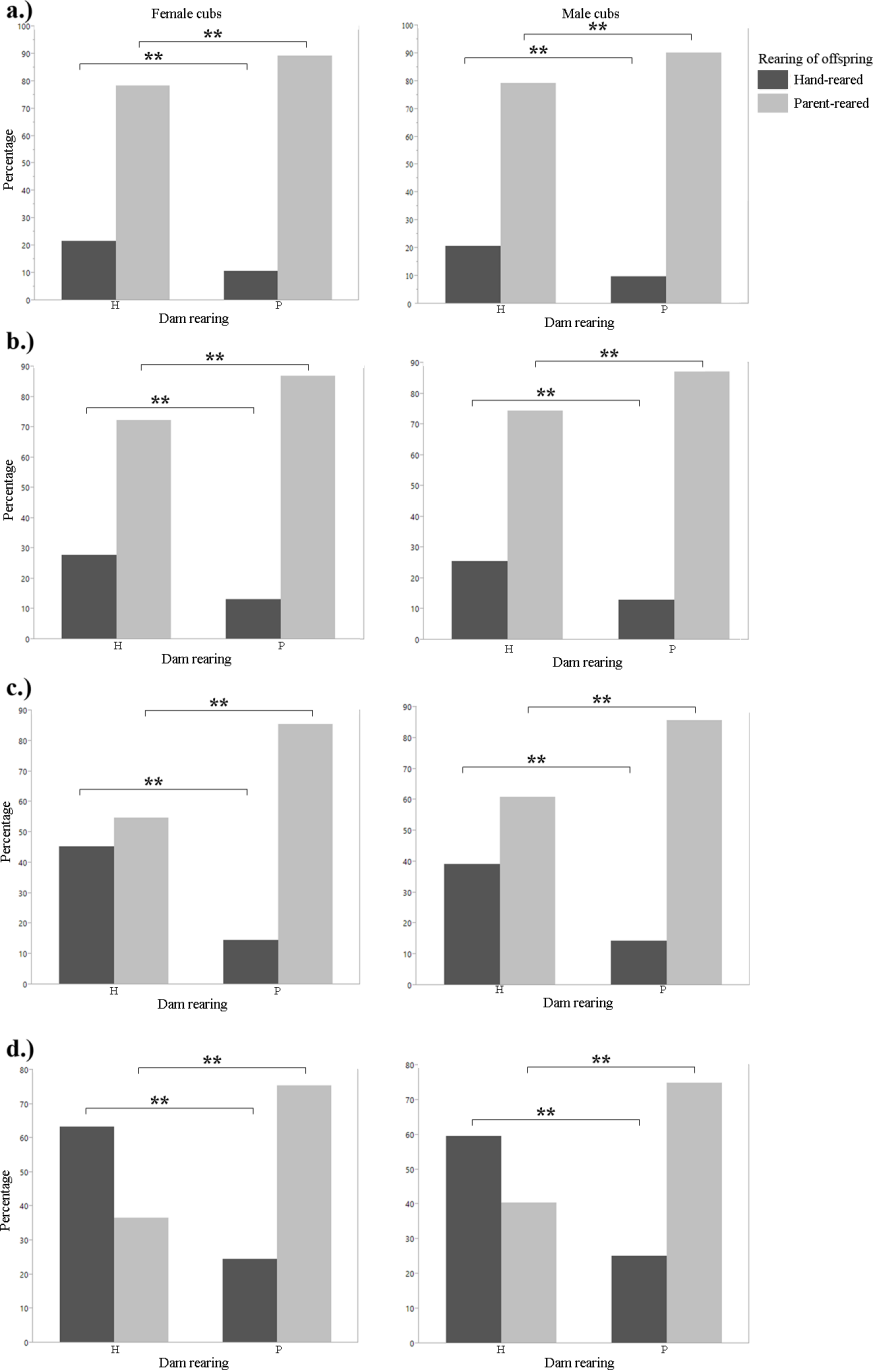
Percentage of female and male offspring that were reared by their dams or hand-reared (1901–2011). A). Siberian tiger B.) snow leopard C). cheetah D). clouded leopard. Fisher's Exact test: *p<0.05; **p<0.01.

We found a significant difference in the number of offspring produced between hand-reared and parent-reared females. Hand-reared females produced fewer offspring than parent-reared females (t-test, F = 7.2978, df = 802, p<0.0001; Fig 1A; Table 1). There was no significant difference for males (t-test, F = 1.4574, df = 686, p = 0.2188; Fig 1A). There was no significant difference in litter size between breeding pairs of different rearing types (Tukey's HSD post-hoc test, F = 1.9889, df = 2044, p = 0.1136). We found no significant difference in age at first reproduction for either females or males between rearing types (t-test, F = 0.4684, df = 360, p = 0.9655 for females; and F = 0.3414, df = 308, p = 0.7525 for males). There was no significant difference in longevity between hand-reared and parent-reared individuals for either gender (t-test, F = 1.6962, df = 839, p = 0.2045 for females; and F = 0.7293, df = 714, p = 0.3915 for males). Infant mortality was not significantly different for either female or male cubs between rearing types (t-test, F = 0.1184, df = 1422, p = 0.7308 for females; and F = 1.1900, df = 1319, p = 0.2755 for males). We found a significant difference in generational rearing between hand-reared and parent-reared dams. Parent-reared dams were significantly more likely to rear their own young than hand-reared dams (Fisher's Exact test, p = 0.0007 for female cubs; and p = 0.0004 for male cubs; Fig 2A; Table 1).

**Table 1:** Summary of results from each analysis.

Species	Sex	Period	Number of offspring		Litter size		Age at first reproduction		Longevity		Infant mortality		Generational rearing	
			(number of young)		(number of young)		(years)		(years)		(occurrence)		(percentage)	
			HR	PR	HR	PR	HR	PR	HR	PR	HR	PR	HR	PR
**Siberian tiger**	males	1901–2011	1.70	2.10	2.20	2.39	5.3	5.4	11.0	9.9	0.50	0.45	22 **	78 **
		2000–2011	0.00 *	0.63 *	0.00	2.30	N/A	N/A	2.0	2.2	0.00	0.00	64	82
	females	1901–2011	1.20 **	2.10 **	2.20	2.39	5.1	5.1	9.2	10.0	0.41	0.40	22 **	78 **
		2000–2011	0.00 *	0.90 *	0.00	2.30	6.0	4.5	0.0 **	3.7 **	0.04	0.08	100	98
**Cheetah**	males	1901–2011	0.07 **	0.00 **	3.18	3.25	5.2	5.3	7.6	7.0	0.00	0.00	40 **	14 **
		2000–2011	0.00	0.00	3.33	3.23	4.7	4.4	3.2 *	2.0 *	0.04	0.04	46 **	17 **
	females	1901–2011	1.33	1.65	3.18	3.25	4.7	5.0	7.4	7.1	0.29	0.26	45 **	14 **
		2000–2011	0.32	0.62	3.33	3.23	4.3	4.2	3.4 **	2.0 **	0.04	0.05	52 **	18 **
**Clouded leopard**	males	1901–2011	1.30	1.30	1.90	1.90	2.7	3.2	9.3	10.0	0.41	0.38	59 **	25 **
		2000–2011	0.42	1.30	1.77	1.87	3.0	2.2	2.2	0.3	0.04	0.04	75 *	45 *
	females	1901–2011	1.30	1.50	1.90	1.90	3.7	3.5	9.4	8.2	0.32	0.42	63 **	23 **
		2000–2011	0.14 **	1.40 **	1.77	1.87	3.0	3.0	2.7	0.3	0.03	0.04	75 *	48 *
**Snow leopard**	males	1901–2011	1.50 **	1.90 **	2.70 *	0.00 *	5.9	5.4	8.1	9.6	0.00	0.00	26 **	13 **
		2000–2011	0.60	0.71	2.00	1.84	4.4	4.7	3.0	3.2	0.05*	0.11 *	17	15
	females	1901–2011	1.30 **	2.00 **	2.70 *	0.00 *	5.3	5.1	10.1	10.0	0.00	0.00	28 **	14 **
		2000–2011	0.00	0.00	2.00	1.84	0.0 **	4.7 **	N/A	N/A	0.08	0.08	24	11

*p<0.05

**p<0.01. *Number of offspring* is the mean number of offspring a female has birthed or a male has sired during their studbook lifetime. *Litter size* is the mean number of individuals born to each dam and sire pair during a single reproductive event. *Age at first reproduction* is the mean age at which individuals gave birth to their first offspring. *Longevity* is the mean number of years an individual lived. *Infant mortality* is the mean occurrence of death before weaning age. *Generational rearing* is the percentage of cubs that were reared by their dams. “N/A” indicates an analysis that could not be completed because there were zero individuals available within the data set.

#### 2000–2011

**Fig 3:**
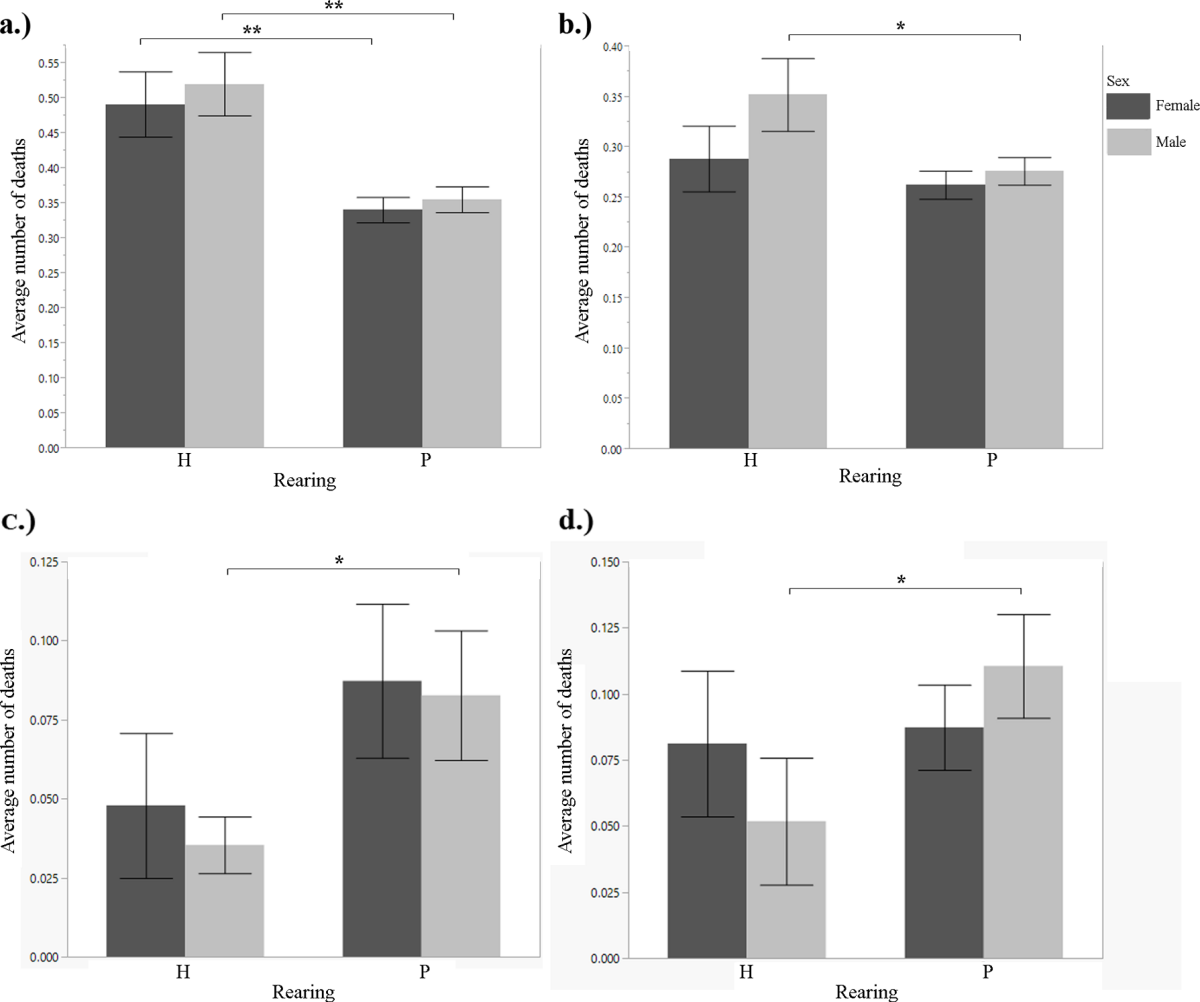
Mean occurrence of death before weaning age, by rearing type. A.) snow leopard (1901–2011) B.) cheetah (1901–2011) C.) Siberian tiger (2000–2011) D.) snow leopard (2000–2011). t-test: *p<0.05; **p<0.01.

**Fig 4:**
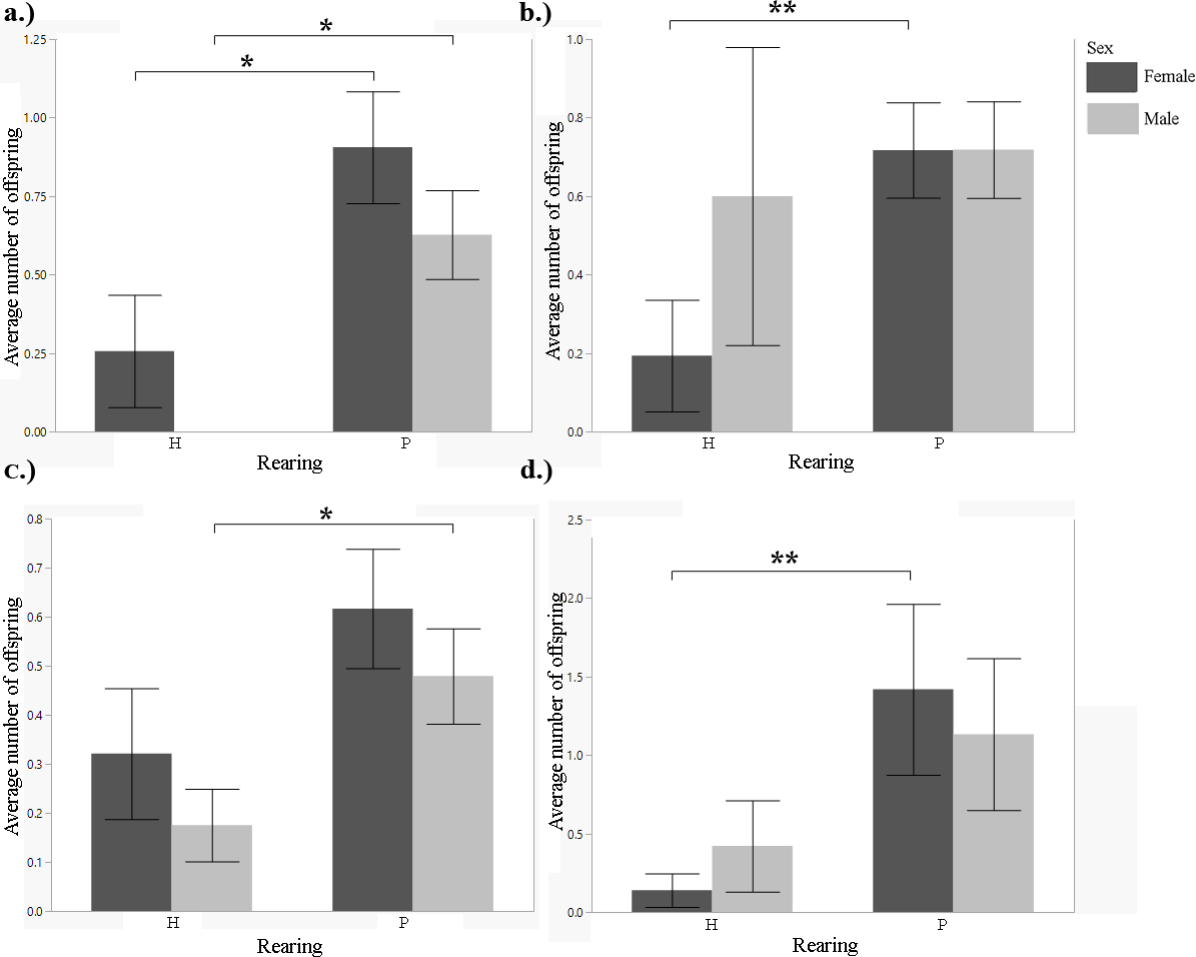
Mean number of offspring a female has birthed or a male has sired during their studbook lifetime, by rearing type (2000–2011). A.) Siberian tiger B.) snow leopard C.) cheetah D.) clouded leopard. t-test: *p<0.05; **p<0.01.

**Fig 5:**
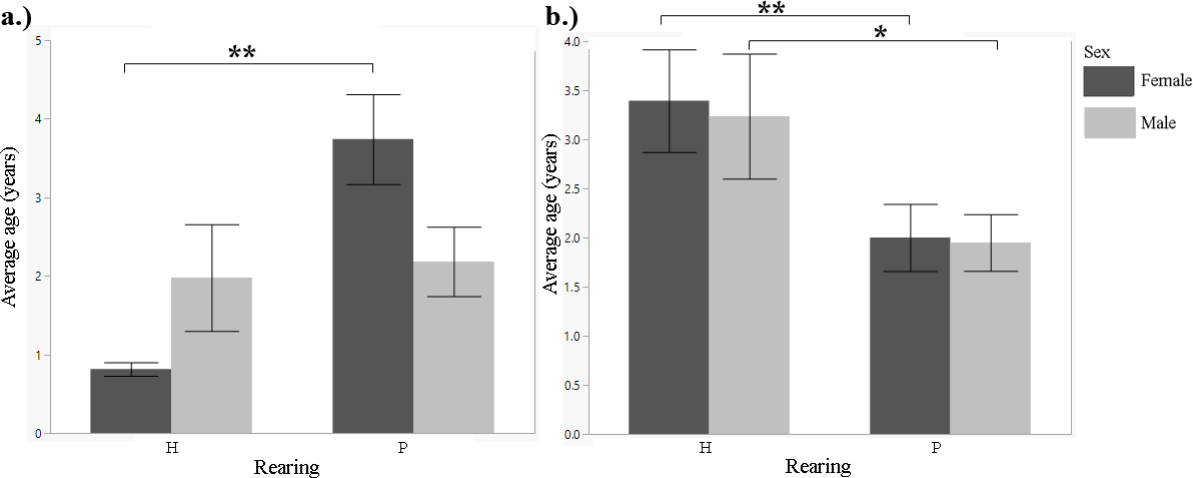
Mean number of years an individual lived, by rearing type (2000–2011). A.) Siberian tiger B.) cheetah. t-test: *p<0.05; **p<0.01.

Whilst infant mortality was significantly lower in hand-reared than in parent-reared male cubs (t-test, F = 2.0153, df = 44, p = 0.0404), it did not differ between rearing types in female cubs (t-test, F = 1.2218, df = 34, p = 0.2471; Fig 3C; Table 1). Hand-reared females and males produced significantly fewer offspring than parent-reared females and males (t-test, F = 2.5996, df = 126, p = 0.0113 for females; F = 3.2952, df = 160, p = 0.001 for males; Fig 4A; Table 1). There was no significant difference in litter size of breeding pairs between rearing types (Tukey's HSD post-hoc test, F = 0.8267, df = 266, p = 0.4386). We found no significant difference in age at first reproduction for females between rearing types (t-test, F = 01.5086, df = 1.2, p = 0.3374). The analysis for males could not be carried out because no hand-reared male tigers reproduced during this time period. There was a significant difference in longevity between hand-reared and parent-reared females (F = 2.4998, df = 20, p = 0.0001; Fig 5A; Table 1). Hand-reared females lived significantly shorter lives than parent-reared females (F = 2.4998, df = 20, p = 0.0001; Table 1). There was no difference in longevity between hand-reared and parent-reared males (F = 0.0528, df = 15, p = 0.8101). We found no significant difference in generational rearing between hand-reared and parent-reared dams (Fisher's Exact test, p = 0.3229 for female cubs; and p = 0.2098 for male cubs).

### Snow leopard

#### 1901–2011

**Fig 6:**
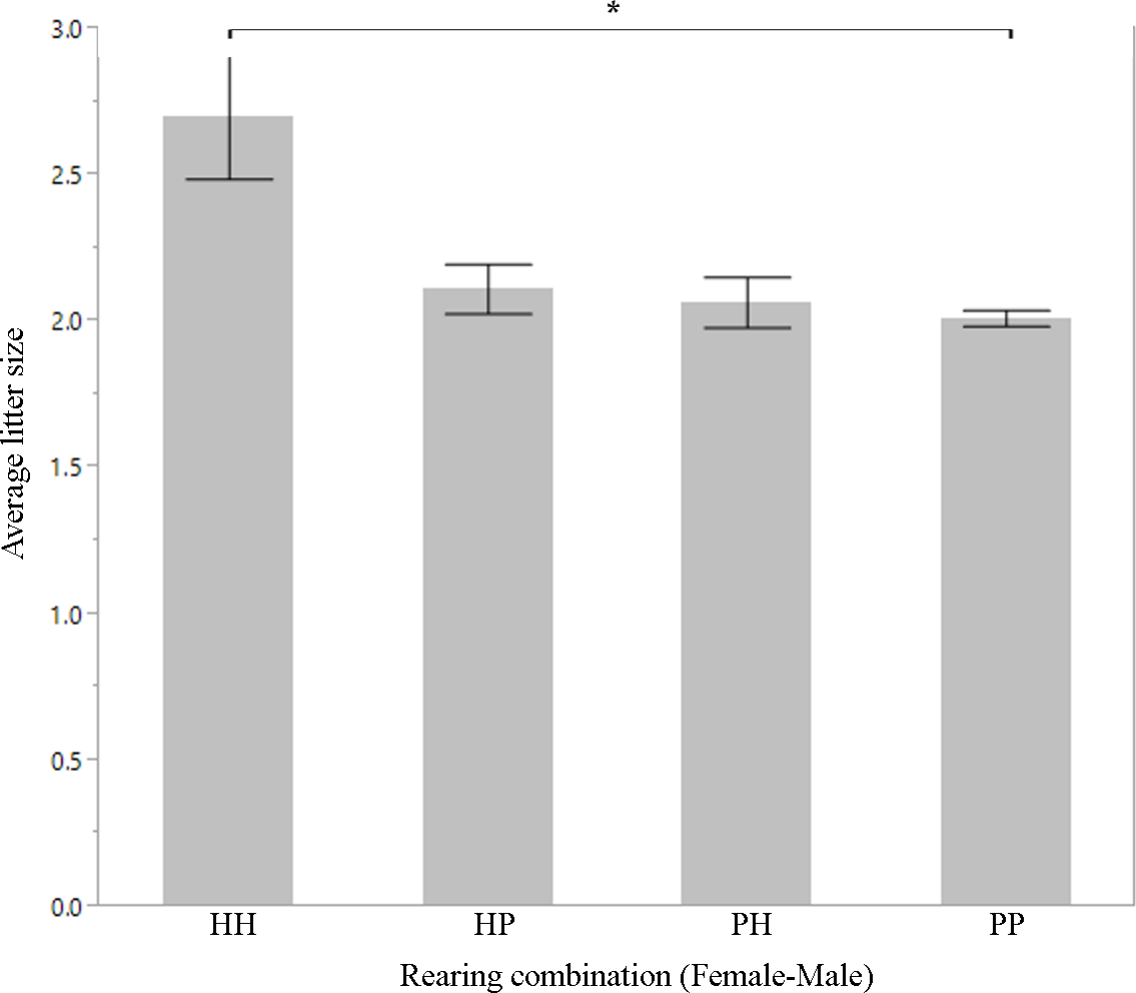
Mean number of individuals born to each dam and sire pair during a single reproductive event for snow leopards 1901–2011. Tukey's HSD post-hoc: *p<0.05; **p<0.01.

We found a significant difference in the number of offspring produced between hand-reared and parent-reared individuals of both genders. Hand-reared females produced fewer offspring than parent-reared females (Mann-Whitney U, z = -3.09055, p = 0.0020; Fig 1B; Table 1). Hand-reared males produced fewer offspring than parent-reared males (Mann-Whitney U, z = -3.17400, p = 0.0015; Fig 1B; Table 1). There was a significant difference in litter size between certain breeding pair combinations (one-way ANOVA, F = 2.7240, df = 1070, p = 0.0431). H-H pairs had larger litters than P-P pairs (Tukey's HSD post-hoc, p = 0.0404; Fig 6; Table 1). There was no significant difference in age at first reproduction for females between rearing types (t-test, F = 0.3068, df = 356, p = 0.5800); and no significant difference in age at first reproduction between hand-reared and parent-reared males (Mann-Whitney U, z = 1.62651, p = 0.1036). There was no significant difference in longevity between hand-reared and parent-reared females or males (t-test, F = 0.0166, df = 506, p = 0.876 for females; F = 2.8595, df = 490, p = 0.0915 for males). There was a significant difference in infant mortality between hand-reared and parent-reared individuals of both genders. Infant mortality was significantly higher in hand-reared than in parent-reared female cubs (Mann-Whitney U, z = 3.12423, p = 0.0018; Fig 3A; Table 1); and significantly higher in hand-reared than in parent-reared male cubs (Mann-Whitney U, z = 3.46199, p = 0.0005; Fig 3A; Table 1). We found that, in both females and males, cubs with parent-reared dams were more likely to be parent-reared themselves (Fisher's Exact test, p = 0.0004 for female cubs; and p = 0.00013 for male cubs; Fig 2B; Table 1).

#### 2000–2011

Hand-reared females produced significantly fewer offspring than parent-reared females (t-test, F = 2.4347, df = 259, p = 0.0064; Fig 4B; Table 1). Males showed no significant difference (t-test, F = 0.0852, df = 217, p = 0.7690; Fig 4B; Table 1). There were no significant differences in litter size between certain breeding pair combinations (one-way ANOVA, F = 0.3786, df = 282, p = 0.7685). There was a significant difference in age at first reproduction for females (t-test, F = 2.0166, df = 44, p = 0.0020; Table 1). Hand-reared females reproduced later in life than parent-reared females. There was no significant difference in age at first reproduction between hand-reared and parent-reared males (t-test, F = 0.1679, df = 2.77, p = 0.5814). Longevity analysis for females could not be completed because there were no hand-reared individuals included in the data set. There was no significant difference in longevity between hand-reared and parent-reared males (t-test, F = 0.0417, df = 9.1, p = 0.6425). There was no significant difference in infant mortality between hand-reared and parent-reared females (t-test, F = 0.0309, df = 28, p = 0.8489). Infant mortality was significantly lower in hand-reared than in parent-reared males (F = 1.6496, df = 26, p = 0.0349; Fig 3D; Table 1). We found no significant differences in generational rearing (Fisher's Exact test, p = 0.3988 for female cubs; and p = 0.3988 for male cubs).

### Cheetah

#### 1901–2011

We found no significant difference in the number of offspring produced between hand-reared and parent-reared females (t-test, F = 1.2190, df = 1654, p = 0.2697; Fig 1C; Table 1). Hand-reared males produced significantly fewer offspring than parent-reared males (t-test, F = 5.5119, df = 1779, p = 0.0018; Fig 1C; Table 1). There were no significant differences in litter size between any combinations of breeding pairs (one-way ANOVA, F = 0.5900, df = 1101, p = 0.6216). There was no significant difference in age at first reproduction between hand-reared and parent-reared individuals (t-test, F = 0.740, df = 304, p = 0.3859 for females; and F = 0.0790, df = 252, p = 0.7789 for males). There was no significant difference in longevity between hand-reared and parent-reared individuals (t-test, F = 0.3732, df = 866, p = 0.5414 for females; and F = 1.2719, df = 877, p = 0.2428 for males). Differences in infant mortality between hand-reared and parent-reared female cubs were not significant (t-test, F = 0.5638, df = 1187, p = 0.4529; Fig 3B). Hand-reared males showed significantly higher infant mortality than parent-reared males (t-test, F = 4.2503, df = 1235, p = 0.0395; Fig 3B; Table 1). We found a significant difference between hand-reared and parent-reared individuals for generational rearing. Parent-reared dams were significantly more likely than hand-reared dams to rear their own offspring (Fisher's Exact test, p<0.0001 for female cubs; and p<0.0001 for male cubs; Fig 2C; Table 1).

#### 2000–2011

**Fig 7:**
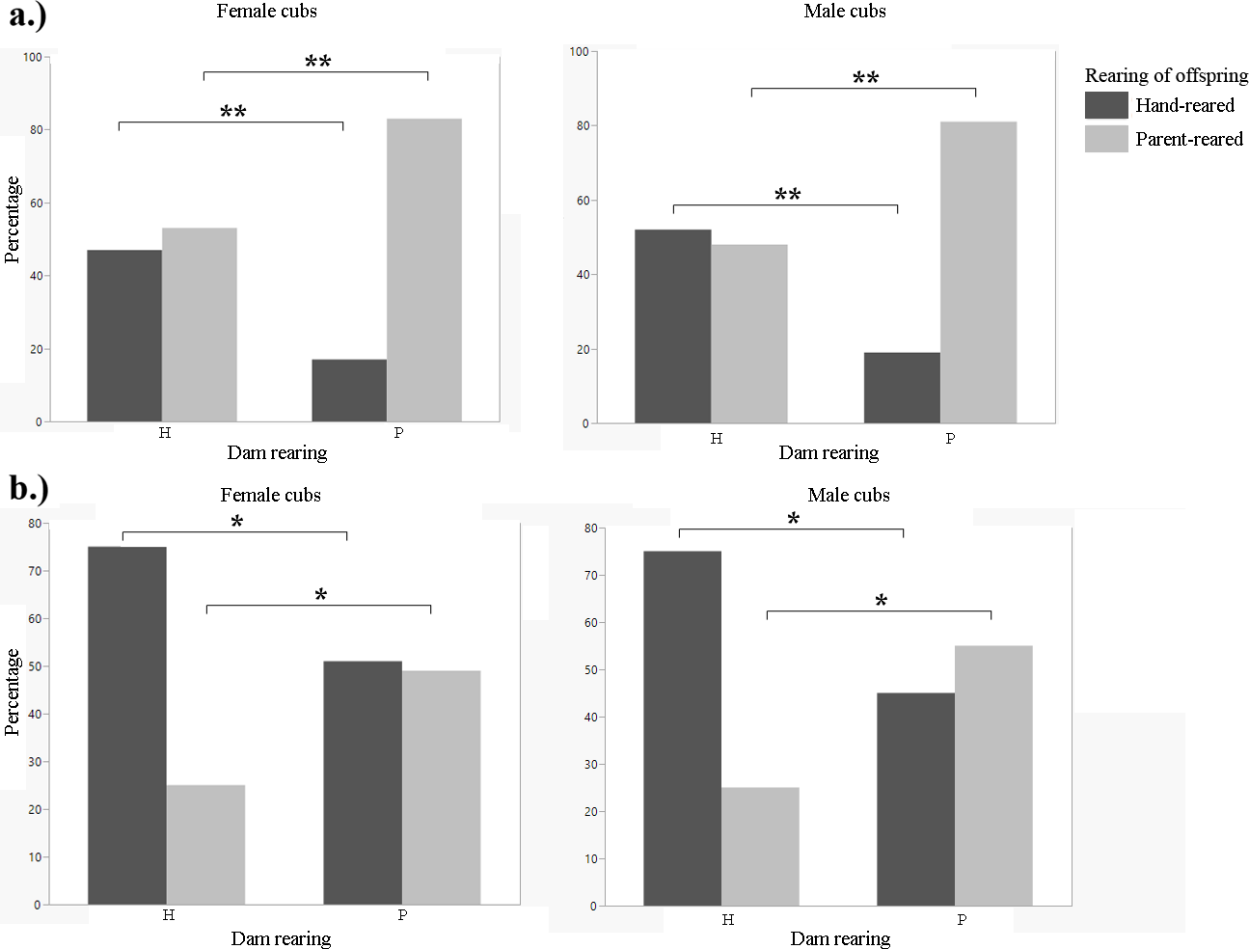
Percentage of female and male offspring that were reared by their dams or hand-reared (2000–2011). A). cheetah B). clouded leopard. Fisher's exact test: *p<0.05; **p<0.01.

We found no significant difference in the number of offspring produced between hand-reared and parent-reared females (t-test, F = 1.7629, df = 428, p = 0.1850; Fig 4C; Table 1). Hand-reared males produced significantly fewer offspring than parent-reared males (t-test, F = 2.9079, df = 473, p = 0.0444; Fig 4C; Table 1). There were no significant differences in litter size between any combinations of breeding pairs (one-way ANOVA, F = 1.9100, df = 3, p = 0.1278). There was no significant difference in age at first reproduction between hand-reared and parent-reared individuals (t-test, F = 0.0215, df = 45, p = 0.8840 for females; and F = 0.1060, df = 34, p = 0.7468 for males). There was a significant difference in longevity between hand-reared and parent-reared individuals. Hand-reared females lived longer than parent-reared females (MWU test, z = 3.04614, p = 0.0023; Fig 5B; Table 1). Hand-reared males lived longer than parent-reared males (MWU test, z = 2.55329, p = 0.0107; Fig 5B; Table 1). Differences in infant mortality between hand-reared and parent-reared cubs were not significant (t-test, F = 0.3890, df = 72, p = 0.4347 for females; F = 0.0357, df = 72, p = 0.8037 for males). We found a significant difference between hand-reared and parent-reared individuals for generational rearing. Parent-reared dams were significantly more likely than hand-reared dams to rear their own offspring (Fisher's Exact test, p<0.0001 for female cubs; and p<0.0001 for male cubs; Fig 7A; Table 1).

### Clouded leopard

#### 1901–2011

We found no significant difference in the number of offspring produced between hand-reared and parent-reared individuals (t-test, F = 0.2866, df = 388, p = 0.5842 for females; and F = 0.0464, df = 414, p = 0.8331 for males; Fig 1D; Table 1). There was no significant difference between breeding pairs of different rearing type with regard to litter size (one-way ANOVA, F = 0.8889, df = 803, p = 0.4464). There was no significant difference in age at first reproduction between hand-reared and parent-reared individuals (t-test, F = 0.1135, df = 70, p = 0.7372 for females; and F = 0.8377, df = 66, p = 0.3634 for males). There was no significance difference in longevity between hand-reared and parent-reared individuals (t-test, F = 1.2941, df = 159, p = 0.2476 for females; and F = 0.6779, df = 171, p = 0.4114 for males). Rates of infant mortality were similar in hand-reared and parent-reared cubs (t-test, F = 1727, df = 262, p = 0.1727 for females; and F = 2789, df = 288, p = 0.5978 for males). We found a significant difference in generational rearing. Parent-reared dams were significantly more likely than hand-reared dams to rear their own offspring (Fisher’s Exact test, p<0.0001 for female cubs; and p<0.0001 for male cubs; Fig 2D; Table 1).

#### 2000–2011

We found a significant difference in the number of offspring produced between hand-reared and parent-reared females (t-test, F = 8.1508, df = 79, p = 0.0055; Fig 4D; Table 1). Parent-reared females produced significantly more offspring than hand-reared females. There was no significant difference for males (t-test, F = 1.7394, df = 66, p = 0.1918; Fig 4D; Table 1). There was no significant difference between breeding pairs of different rearing type with regard to litter size (one-way ANOVA, F = 2.1620, df = 3, p = 0.0991). There was no significant difference in age at first reproduction between hand-reared and parent-reared individuals (t-test, F = 0.0208, df = 9, p = 0.8886 for females; and F = 3.3672, df = 8, p = 0.1038 for males). There was no significance difference in longevity between hand-reared and parent-reared individuals (t-test, F = 6.6241, df = 2, p = 0.1236 for females; and F = 2.8095, df = 3, p = 0.1923 for males). Rates of infant mortality were similar for hand-reared and parent-reared cubs (t-test, F = 0.2286, df = 9, p = 0.6440 for females; and F = 0561, df = 8, p = 0.8187 for males). We found a significant difference in generational rearing. Parent-reared dams were significantly more likely than hand-reared dams to rear their own offspring (Fisher’s Exact test, p = 0.0386 for female cubs; and p = 0.0232 for male cubs; Fig 7B; Table 1).

## Discussion

The results of this study draw into question the practice of hand-rearing offspring in the four studied large felid species, as hand-reared individuals differed from parent-reared individuals in some of the variables tested. In interpreting the results, it is important to note that a lack of reproductive success in an individual may be attributed to a lack of opportunity for that individual to reproduce. This can for example occur as a result of limited access to mates or infertility. Access to mates is controlled by the SSP or EEP coordinator, who makes breeding recommendations on the basis of inbreeding coefficients and mean kinship values [4]. However, since all of the study species are part of SSPs and EEPs, it was assumed that hand-reared and parent-reared individuals had equal levels of fertility and equal access to mates.

In the pursuit of a “clean” data set, those individuals that did not fall within the rearing categories H and P or gender categories 0 and 1 were not included in the analysis. Between all four species of large felids, 21.5–49.5% of the total data set were excluded due to these parameters. Cheetahs had the highest exclusion percentage at 49.5%, because many of them were wild-caught, categorizing the dam and sire rearing types as “wild.” As a result of these exclusions, the data set analysed in this study consisted of at least second-generation captive born individuals, and onwards. This allowed for better analysis of the factors affecting large felids solely within zoological institutions without any confounding variables that may be added from a life lived partly in the wild. Collecting data from studbooks yields a robust sample size, but it does not provide any background information on individuals. Studbooks did not show what environmental or behavioural factors were present, which limits the conclusions that can be drawn from this type of research. For this study, it was assumed that any such factors would have affected hand-reared and parent-reared individuals equally.

Individuals with blank data points for any of the variables (number of offspring, litter size, age at first reproduction, longevity, infant mortality and generational rearing) were not included in certain analyses. Individuals were excluded on a per-analysis basis. This means that while some individuals were excluded from one analysis, they could be included in others depending upon which data points were present. The number of individuals that were “lost to follow up” ranged from 25.0 to 35.7%, indicating a weakness in studbook record keeping. Weaknesses in record keeping are a hindrance to studbook research. While it may not be important for an individual zoological institution to continue to track an animal after it was transferred to another collection, it would be valuable for SSPs or EEPs to have this information.

In the 1901–2011 dataset, hand-reared female Siberian tigers, female and male snow leopards, and male cheetahs produced fewer offspring than parent-reared individuals of the same species and gender. When looking at a subset of data from 2000–2011, hand-reared male Siberian tigers, and female and male clouded leopards produced fewer offspring than parent-reared individuals. Analysis of the studbook data alone cannot single out the cause of these differences. Several large felid species show oestrus behaviours such as rubbing, rolling, vocalizations, urine spraying and sniffing [31, 32]. There is a possibility that hand-reared females may fail to display these oestrus cues altogether, or display them at inappropriate times, which could result in a lack of breeding. Conversely, hand-reared males may not interpret these cues as a signal to breed. Lower offspring numbers in hand-reared individuals may also result from a zoo's management strategy. Annual breeding and transfer plans determine how many breeding pairs are required and which individuals will be allowed to breed during the year [33, 34]. Individuals are selected by SSP/EEP coordinators based on age, relative genetic value, relatedness of individuals, logistics, health status and behaviour. Hand-reared individuals may not be given the same opportunities to breed as parent-reared individuals due to assumed or observed deficits in any of these categories. Appropriate social housing can improve animal welfare by promoting the expression of wild behaviours [35, 36] and affect reproductive success [37, 38, 39]. However, it is unclear what effects certain housing strategies can have on breeding success. Some investigations have shown that constant exposure to a potential mate outside of oestrus can promote successful reproduction in felids [35, 40, 41], while others have shown continuously housing cats together can have a negative effect on breeding success [42, 43]. For male cheetahs specifically, the formation and maintenance of coalitions has been shown to improve chances of survival and reproductive success [39, 44, 45]. It is also possible that other factors not examined in this study could contribute to the differences in number of offspring, such as enclosure design, keeper interactions or husbandry schedules, although they should have affected hand-reared and parent-reared individuals in a similar way.

The snow leopard was the only species that showed a difference in litter size between breeding pairs for both the 1901–2011 and the 2000–2011 data subsets. H-H pairs had larger litters than P-P pairs, with a trend for H-H pairs to have larger litters than P-H pairs as well. It is difficult to pinpoint the factors that affect litter size. In island foxes (*Urocyon littoralis*), differences in litter size can be attributed to male age and wind exposure [46], while red wolves (*Canis rufus*) have smaller litters as they age [47]. Factors such as these could not be analysed in this study as studbook data do not provide such information: only differences in rearing type were accounted for. In this study, rearing type did not affect litter size for three out of the four species examined. More data are required to determine why snow leopards may differ in this regard and what factors led to H-H pairs having had larger litters than P-P and P-H pairs.

There was no significant difference between rearing types for age at first reproduction for any of the species from 1901–2001. The dataset from 2000–2011 showed that hand-reared female snow leopards reproduced later in life than parent-reared females. It is difficult to attribute any concrete factors that may have contributed to this difference. Past studies have shown that parent-reared female gorillas conceived one year earlier than hand-reared females [20], however we cannot draw any conclusions from the studies relating to the felid species analysed here. In captivity, breeding opportunities can be artificially manipulated. How soon an animal can breed will depend on when it is given the opportunity. If an animal is genetically over-represented, unable to be paired with a genetically suitable mate or incompatible with its breeding partner, then breeding may be delayed.

In the 2000–2011 dataset, hand-reared female Siberian tigers and hand-reared female and male cheetahs lived shorter lives than their parent-reared counterparts. All other species showed no significant differences in longevity between rearing types. Parental rearing has been known to have nutritional, developmental and behavioural benefits for the offspring [9, 17]. Diet, accommodation and general husbandry standards could affect longevity, but these factors were not studied here. Since the results of this analysis were mostly insignificant, it would appear that these factors affect both hand-reared and parent-reared individuals equally.

Infant mortality was significantly higher for hand-reared female and male snow leopard cubs and male cheetah cubs in the 1901–2011 dataset; and significantly lower for hand-reared male Siberian tiger and snow leopard cubs in the 2000–2011 dataset. In cheetahs, tigers and domestic cats, large litter sizes have been attributed to lower infant survival rates [48, 49], and studies on captive golden lion tamarins (*Leontopithecus rosalia*) have shown that parental rearing increases offspring survival rates [50]. An animal classified as hand-reared at this stage would already be in human care, meaning the death of an infant would not be the fault of the mother. Infant survival can decrease due to hand-rearing [51]. With Siberian tigers, staff experience with breeding and rearing tigers can influence both reproductive success and cub survival [51]. The 2000–2011 results may indicate an improvement in staff experience, as hand-reared cubs had a lower infant mortality rate than parent-reared cubs. Cubs that are neglected or abandoned by their mothers may have underlying health problems that zoo staff might be unaware of. In this scenario, even a well-equipped facility with knowledgeable staff would not be able to ensure cub survival.

All species in the 1901–2011 analysis showed a significant relationship between the rearing of a mother and subsequent rearing of her offspring. From 2000–2011, only cheetahs and clouded leopards showed a significant difference. Parent-reared females were significantly more likely to raise their own cubs than hand-reared females. This has been seen in gorillas as well. Ryan et al. (2002) found that parent-reared female gorillas were more likely to become nurturing mothers themselves and rear their own young. Studies on various callitrichid primate species have shown that an individual's early social experiences may impact its ability to rear its own infants [52]. The husbandry guidelines for clouded leopards indicate that several facilities choose to hand-rear their cubs [8]. This conscious decision could have skewed the results for generational rearing of clouded leopards, depending on which facility they were housed in. However, the results showed that parent-reared females were more likely to rear their own cubs than hand-reared females, and no distinction is made in the husbandry manual about rearing cubs depending on the rearing of their parents. Hand-rearing can occur for several reasons, two of which the clouded leopard husbandry manual lists as aggression towards cubs or lack of interest in rearing cubs [8]; it is yet to be determined if these behaviours are seen more often in parent-reared or hand-reared dams, or if both rearing types are equally likely to display these behaviours, resulting in hand-rearing of their cubs. The difference between the 1901–2011 and the 2000–2011 data subsets indicated that husbandry or other factors may have improved over time, allowing more females to raise their own cubs. However, which factors have changed and how cannot be determined without further research. The finding that hand-reared mothers were significantly less likely to rear their own young, combined with indications that hand-reared individuals of some species also produced a significantly lower number of offspring, implies a potential “extinction vortex” within managed conservation breeding programmes. Hand-reared individuals produce fewer offspring, and the offspring produced will likely be hand-reared themselves and go on to produce fewer offspring. This breeding deficit and pattern could be detrimental to SSPs and EEPs for highly threatened species.

Hand-rearing can have developmental and nutritional impacts on the individual. There are many factors such as reproductive behaviour, rearing ability and chances of offspring survival that cannot be studied through studbook data alone. The aim of this study was to investigate the reproductive success of hand-reared felids compared to that of parent-reared individuals. The analysis of the data has begun to paint a picture of how hand-rearing can affect reproductive success, but this should only be the beginning. Past research has shown that infant primates that do not receive early socialization can show abnormal behaviour [53, 54], abnormal reproductive behaviour [18, 23, 55, 56], and insufficient maternal behaviour [4]. For captive cheetahs, imprinting on humans can cause behavioural problems [7]. Hand-reared Iberian lynx (*Lynx pardinus*) cubs were found to have more contact time with keepers, and this can increase occurrences of imprinting [57]. While keepers are also present around parent-reared individuals during daily husbandry routines, past research has found that parent-reared black rhinos (*Diceros bicornis*) and maned wolves (*Chrysocyon brachyurus*) had less affinity for their keepers than hand-reared counterparts [58]. Hand-rearing has been shown to have an effect on the ability of an individual to rear their offspring, which affects chances of offspring survival. Studies on callitrichid species have shown that an individuals' early social experience may affect its ability to rear infants in the future [52]. A study on golden lion tamarins showed that parent-rearing significantly increased offspring survival rates [50]. Hand-rearing can have nutritional impacts on an individual as well. Hand-reared felids have been observed to develop hair loss at six to eight weeks, and this is thought to be caused by a deficiency in diet [17]. Cheetah cubs reared by their mothers are better behaviourally adjusted and have a reduced chance of suffering from nutritional deficiencies [17]. Overall, more research is required to determine how some of these factors, observed in other non-felid species, manifest in the felids studied here in order to deepen the knowledge already obtained by a limited amount of felid research on hand-rearing.

Further research is also needed to determine how the variables analysed in this study relate to one another and how this may affect the integrity of SSPs and EEPs. For example, male hand-reared snow leopards included in the 2000–2011 dataset lived an average of three years, while the average age at first reproduction in this species was close to five years. This could lead to fewer offspring produced simply because the reproductive lifespans of the individual is decreased. These relationships were not analysed in this study, but would be a vital component for determining how hand-rearing and hand-rearing practices relate to reproductive success.

The results of our study show differences between hand-reared and parent-reared individuals in different reproductive variables. While it would not affect SSPs or EEPs to hand-rear animals destined for education and outreach programmes, the differences in number of offspring and generational rearing patterns suggest that hand-rearing individuals that are part of the captive breeding programmes could be detrimental to the programmes’ integrity. Further research should be done on the factors affecting breeding success in large felids, so that more effective hand-rearing policies can be put into practice.

## Conclusions

1. Hand-rearing appears to negatively affect the number of offspring, litter size, infant mortality and generational rearing for some of the large felid species in this study. The exact factors causing these differences remain to be determined.
2. The results demonstrated that hand-reared Siberian tigers produced fewer offspring; and parent-reared dams were more likely to rear their own offspring. Hand-reared snow leopards produced fewer offspring and had higher infant mortality. H-H pairs produced larger litters; and parent-reared dams were more likely to rear their own young. Male hand-reared cheetahs produced fewer offspring and had higher infant mortality. Parent-reared dams were more likely to rear their own progeny. Parent-reared clouded leopard dams were more likely to rear their own offspring.
3. Management of captive populations may affect number of offspring, age at first reproduction, longevity, infant mortality and generational rearing. The exact effects of individual zoo management strategies on any of these variables have yet to be determined.
4. Our finding that hand-reared mothers are significantly less likely to rear their own young, combined with indications that hand-reared individuals of some species also produced a significantly lower number of offspring, implies a potential “extinction vortex” within managed conservation breeding programmes.

## Supporting Information

S1 Table SQL query for number of offspring, age at first reproduction, longevity, infant mortality, and generational rearing. (PDF)

S2 Table SQL query for litter size. (PDF)
