# Supplementary material for: Effects of Hand-Rearing on Reproductive Success in Captive Large Cats Panthera tigris altaica, Uncia uncia, Acinonyx jubatus and Neofelis nebulosa
Source: PLoS One. 2016 May 23;11(5):e0155992. doi: 10.1371/journal.pone.0155992 (PMC4877043; doi:10.1371/journal.pone.0155992)
Supplement: S1 Table — (PDF) [file pone.0155992.s001.pdf]

S1 Table. SQL query for number of offspring, age at first reproduction, longevity, infant mortality, and generational rearing

```
SELECT
   m.STUD_ID,
   m.SEX,
   m.REARING,
   m.BDATE,
   death.TRAN_DATE AS date_of_death,
   ROUND((death.TRAN_DATE-m.BDATE) / 365, 2) AS age_at_death,
   (SELECT min(child.BDATE) FROM MASTER child WHERE child.DAM_ID =
m.STUD_ID OR child.SIRE_ID = m.STUD_ID) AS date_of_first_child,
   ROUND(( (SELECT min(child.BDATE) FROM MASTER child WHERE
child.DAM_ID = m.STUD_ID OR child.SIRE_ID = m.STUD_ID) - m.BDATE) /
365, 2) AS age_of_first_reproduction,
   (SELECT count(*) FROM MASTER WHERE DAM_ID=m.STUD_ID OR
SIRE_ID=m.STUD_ID) AS number_of_offspring,
   dam.REARING AS dam_rearing,
   sire.REARING AS sire_rearing,
   dam.REARING + sire.REARING AS rearing_combo
FROM
   ((MASTER m
   LEFT JOIN MASTER dam ON m.DAM_ID = dam.STUD_ID)
   LEFT JOIN MASTER sire ON m.SIRE_ID = sire.STUD_ID)
   LEFT JOIN MOVES death ON (death.STUD_ID=m.STUD_ID AND
death.TRAN_CODE='BA')
WHERE
```

```
m.SEX IN (0,1)

AND m.REARING IN ('H','P')

AND dam.REARING IN ('H','P')

AND sire.REARING IN ('H','P')
```
