# Supplementary material for: Effects of Hand-Rearing on Reproductive Success in Captive Large Cats Panthera tigris altaica, Uncia uncia, Acinonyx jubatus and Neofelis nebulosa
Source: PLoS One. 2016 May 23;11(5):e0155992. doi: 10.1371/journal.pone.0155992 (PMC4877043; doi:10.1371/journal.pone.0155992)
Supplement: S2 Table — (PDF) [file pone.0155992.s002.pdf]

S2 Table. SQL query for litter size.

```
SELECT

      m.BDATE AS litter_event_date,

      m.DAM_ID,

      dam.REARING AS dam_rearing,

      sire.REARING AS sire_rearing,

      dam.REARING + sire.REARING AS rearing_combo,

      count(*) AS litter_size

FROM

      (MASTER m

      INNER JOIN MASTER dam ON m.DAM_ID=dam.STUD_ID)

      INNER JOIN MASTER sire ON m.SIRE_ID=sire.STUD_ID

WHERE

      dam.REARING IN ('H','P')

      AND sire.REARING IN ('H','P')

      AND m.SEX IN (0,1)

      AND dam.SEX = 0

      AND sire.SEX = 1

GROUP BY

      m.BDATE, m.DAM_ID, dam.REARING, sire.REARING
```
